# Ethosomal Gel for Topical Administration of Dimethyl Fumarate in the Treatment of HSV-1 Infections

**DOI:** 10.3390/ijms24044133

**Published:** 2023-02-18

**Authors:** Mariaconcetta Sicurella, Walter Pula, Karolina Musiał, Katarzyna Cieślik-Boczula, Maddalena Sguizzato, Agnese Bondi, Markus Drechsler, Leda Montesi, Elisabetta Esposito, Peggy Marconi

**Affiliations:** 1 Department of Environmental Sciences and Prevention, University of Ferrara, I-44121 Ferrara, Italy; 2 Department of Chemical, Pharmaceutical and Agricultural Sciences, University of Ferrara, I-44121 Ferrara, Italy; 3 Faculty of Chemistry, University of Wroclaw, Joliot-Curie 14, 50-383 Wroclaw, Poland; 4 Bavarian Polymer Institute (BPI) Keylab “Electron and Optical Microscopy”, University of Bayreuth, D-95440 Bayreuth, Germany; 5 Department of Life Sciences and Biotechnology, University of Ferrara, I-44121 Ferrara, Italy

**Keywords:** dimethylfumarate, cryogenic transmission electron microscopy, HSV-1, infection control, in vitro diffusion

## Abstract

The infections caused by the HSV-1 virus induce lesions on the lips, mouth, face, and eye. In this study, an ethosome gel loaded with dimethyl fumarate was investigated as a possible approach to treat HSV-1 infections. A formulative study was conducted, evaluating the effect of drug concentration on size distribution and dimensional stability of ethosomes by photon correlation spectroscopy. Ethosome morphology was investigated by cryogenic transmission electron microscopy, while the interaction between dimethyl fumarate and vesicles, and the drug entrapment capacity were respectively evaluated by FTIR and HPLC. To favor the topical application of ethosomes on mucosa and skin, different semisolid forms, based on xanthan gum or poloxamer 407, were designed and compared for spreadability and leakage. Dimethyl fumarate release and diffusion kinetics were evaluated in vitro by Franz cells. The antiviral activity against HSV-1 was tested by plaque reduction assay in Vero and HRPE monolayer cells, while skin irritation effect was evaluated by patch test on 20 healthy volunteers. The lower drug concentration was selected, resulting in smaller and longer stable vesicles, mainly characterized by a multilamellar organization. Dimethyl fumarate entrapment in ethosome was 91% *w*/*w*, suggesting an almost total recovery of the drug in the lipid phase. Xanthan gum 0.5%, selected to thicken the ethosome dispersion, allowed to control drug release and diffusion. The antiviral effect of dimethyl fumarate loaded in ethosome gel was demonstrated by a reduction in viral growth both 1 h and 4 h post-infection. Moreover, the patch test demonstrated the safety of the ethosomal gel applied on the skin.

## 1. Introduction

The human pathogen herpes simplex virus type 1 (HSV-1) can induce frequent recurrent infections especially in the orofacial zone, resulting in the formation of epidermal lesions inside and around the mouth, in nose, as well as in other body districts, such as fingers [1,2,3]. In addition, HSV-1 affects nearly every ocular tissue, including the corneal epithelium, stroma, or endothelium, leading to herpes stromal keratitis and herpes endotheliitis, which can eventually result in loss of vision due to corneal scar formation and neovascularization, while in the case the retina infection, HSV-1 can lead to acute retinal necrosis [4,5,6,7]. Regrettably, HSV-1 is never completely eradicated from the host since it can establish latency, maintaining a relatively quiescent state during which the viral genome is retained without producing virus particles, while it can reactivate, causing recurrent diseases in response to certain stressor, evading host antiviral innate immune responses [8]. The current standard of care in the treatment of HSV-1 infections is based on topical antivirals, among which acyclovir is the first-line drug, beyond topical corticosteroids, in the case of stromal keratitis [9]. However, despite their efficacy, the long-term use of antivirals can induce drug resistant virus strains, while corticosteroids can cause serious side effects. In this respect the treatment of HSV-1 infections represents an unmet need, requiring alternate efficacious drugs [9].

Notably, it has been demonstrated that the course of herpetic keratitis can be improved with fumaric acid ester treatment [10,11]. Dimethyl fumarate (DMF) is a fumaric acid esters derivate that can be considered as a pleiotropic drug, possessing immuno-modulatory, anti-inflammatory, and antioxidant properties that make it efficacious in many conditions, including inflammatory, degenerative, neoplastic, and cardiovascular diseases [12]. Indeed, DMF is used in the systemic treatment of psoriasis with a safe profile for long-term therapy [13,14], it has been approved by the Food and Drug Administration in the USA to treat relapsing-remitting multiple sclerosis [15], has potential applications to limiting HIV disease progression [16], a strong potential in eye pathologies, suggesting its use in several ophthalmological context [17,18], while it is also able to improve wound healing under diabetic conditions [19,20].

Despite the effectiveness, good tolerability and bioavailability of orally administered DMF, some adverse gastrointestinal effects (i.e., diarrhea, vomiting and nausea) have been described [12]. To treat local pathologies, such as HSV-1 infections, affecting orofacial or eye regions, a topical administration should be preferable with respect to the systemic route, due to many pharmacokinetic and pharmacodynamic aspects, including the possibility to use a lower drug dosage, and to deliver the drug directly on the affected tissue, thus reducing systemic side effects [9]. On the other hand, DMF topical use has not been thoroughly investigated, probably due its possible side effects and to a scarce knowledge about its safety profile. Thus, in this respect, cytotoxicity studies, as well as patch tests, are necessary to explore DMF suitability in the treatment of cutaneous, oromucosal or ophthalmic pathologies [12]. In addition, semisolid formulation suitable to deliver DMF directly in the affected body district (e.g., lips or eye) should be specifically designed.

A recent ex vivo and in vivo evaluation has demonstrated the possibility to load DMF in a nano-vesicular phosphatidylcholine (PC) based gel suitable for cutaneous administration. The selection of DMF safe dosage enabled to obtain a formulation potentially effective in the treatment of wounds caused by diabetes mellitus or peripheral vascular diseases [20].

The dispersion of PC in water under specific conditions can generate several lyotropic liquid crystalline phases, possessing interesting features as drug delivery systems, such as liposomes or ethosomes (ETHO). ETHO can be described as colloidal dispersions in which the disperse phase is constituted of PC organized as multilamellar vesicles, while the dispersing phase is an ethanol/water mixture (ethanol 20–45%) [21,22]. These nano-vesicular systems can entrap hydrophilic and lipophilic drugs, controlling their release and promoting their transdermal delivery [23]. The presence of PC and ethanol confers softness and thermodynamical stability to the vesicles, while the penetration enhancer properties of ETHO components promote their passage through the biological barriers, allowing the intracellular delivery of the entrapped drugs [24,25,26].

In this respect, in the present study an ETHO based formulation is proposed as a DMF delivery system to treat HSV-1 infections. To elucidate the influence of DMF loading on ETHO, their physico-chemical features were investigated, evaluating size, morphology, entrapment capacity, distribution of functional groups and chemical structure evolution. Moreover, an ETHO hydrogel was specifically designed to obtain a safe biomaterial suitable for topical application. Indeed, due to their consistency and high-water content, hydrogels can be comfortably applied on biological surfaces, such as the skin, eye and mucosae [27]. Notably, some authors demonstrated that various topical infectious diseases can be treated using nanovesicle hydrogels that can longer sustain drug release with respect to the corresponding plain nanovesicles, prolonging the contact time with the biological surface [28,29,30]. Particularly, the treatment of ocular infectious diseases requires frequent eye drop administrations, possibly resulting in drug resistance and also in decrease of patient compliance [31]. Therefore, in order to minimize precorneal drainage and to increase drug bioavailability, thickening agent can be added to eye drop forms [32]. To this aim, both xanthan gum (x-gum) and poloxamer 407 (p-407) have been evaluated, being able to produce biocompatible and biodegradable hydrophilic gels, suitable for administration on skin, lips, and eye. X-gum is a natural heteropolysaccharide, constituted of d-glucose, d-mannose, d-glucuronic acid, acetal-linked pyruvic acid, and O-acetyl repeating units, employed in many fields, including food, cosmetics, and pharmaceutical applications, being able to produce transparent and stable gels upon dispersion in water [33]. P-407 is a non-ionic poly(oxyethylene)poly(oxypropylene) (PEO-PPO) block copolymer, mainly employed in pharmaceutics, due to its thermo-reversible character under dispersion in water [34]. Indeed, p-407 micellar solution is suitable for cutaneous and mucosal administration, due to its liquid state, while in contact with body temperature it assumes a semisolid consistency, allowing to control drug release [35]. The release and permeability profiles of DMF loaded in the selected gel were evaluated in vitro by Franz cells. Furthermore, an in vivo irritation test was conducted to verify ETHO gel safeness, while the antiviral activity of DMF loaded in ETHO or in ETHO gel was studied, evaluating their inhibitory capacity on plaque formation of HSV-1 in Vero (African green monkey kidney) and HRPE (Human Retinal Pigment Epithelial Cells)monolayer cells.

## 2. Results

### 2.1. Preparation of Ethosomes

ETHO were designed as lipid colloidal systems suitable for DMF delivery through the skin and mucosae. ETHO preparation was simply performed by adding water under stirring to PC ethanol solutions [26]. To load DMF in ETHO, the drug (0.5 or 1 mg/mL) was solubilized in PC ethanol solutions before the addition of water (Table 1).

Both loaded and unloaded ETHO appear as whitish homogeneous dispersions, free from separation phenomena. A preformulative study was conducted to evaluate the effect of DMF on vesicle size distribution and stability.

### 2.2. Characterization of Ethosomes

#### 2.2.1. Size Distribution

Size distribution parameters of ETHO measured by Photon Correlation Spectroscopy (PCS) are reported in Table 2. Mean diameters of the vesicles ranged between 212 and 231 nm, the presence of DMF-induced a slight size increase, particularly DMF 1 mg/mL led to the formation of a low represented population of big vesicles with diameter larger than 4 μm. Dispersity indexes were anyway lower than 0.25.

**Figure 1:**
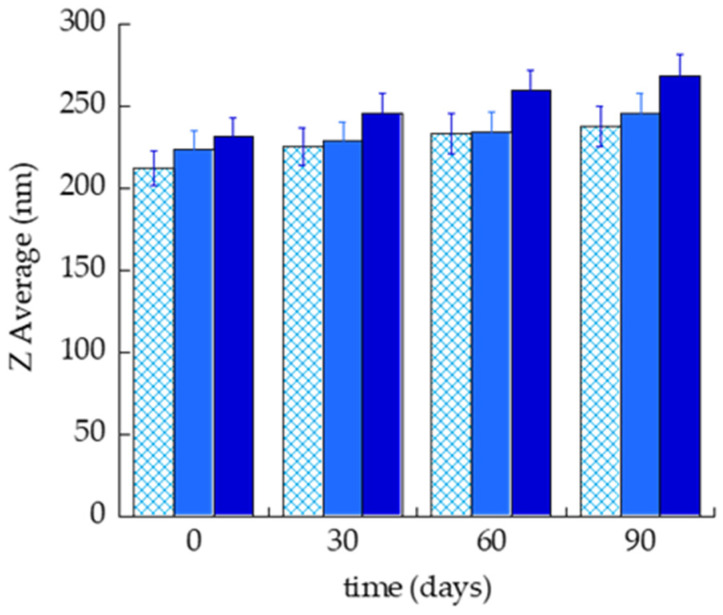
Variation of Z Average of ETHO (criss-cross), ETHO-DMF_0.5_ (light blue) and ETHO-DMF_1.0_ (blue), as measured by PCS for 3 months on samples stored at 22 °C.

In order to detect the vesicle stability, PCS measurements were performed monthly for 3 months. As depicted in Figure 1, vesicle mean diameters underwent a modest increase, reaching 268 nm in the case of ETHO-DMF_1.0_. SIR values, calculated to determine the effect of DMF content on size stability (Table 2), suggest a longer stability in the case of ETHO-DMF_0.5_, followed by ETHO and ETHO-DMF_1.0_. For this reason, ETHO-DMF_0.5_ was selected for further experiments.

#### 2.2.2. Morphology

**Figure 2:**
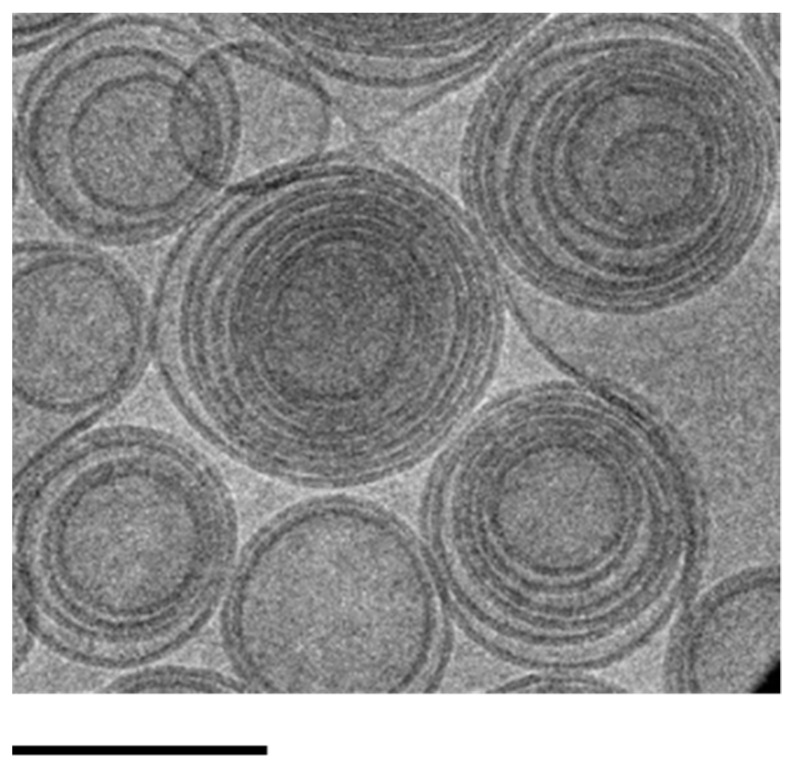
Cryo-TEM image of ETHO-DMF_0.5_. The bar corresponds to 100 nm.

The morphology of ETHO-DMF_0.5_ was investigated by cryogenic transmission electron microscopy (cryo-TEM). The micrograph reported in Figure 2 shows the fingerprint like structure typical of ETHO, reflecting the PC supramolecular organization in double layered multilamellar spherical vesicles, as well as unilamellar, and multi vesicular vesicles [21].

#### 2.2.3. Fourier-Transform Infrared Spectroscopy (FTIR) Studies

FTIR studies were conducted to structurally characterize ETHO. Indeed, this technique provides significant information on molecular structure, specifically on the chemical functional groups of organic compounds by identifying the vibrational signatures related to specific types of chemical bonds [36]. Particularly, FTIR studies were conducted on ETHO and ETHO-DMF_0.5_ prepared using D_2_O instead of H_2_O. The use of D_2_O did not affect the macroscopic aspect of samples, nor the size distribution of the vesicles. Indeed, PCS analyses revealed Z Average values of 229.2 ± 15.1 nm and 208.3 ± 7.2 for ETHO and ETHO-DMF_0.5_, respectively, while dispersity values were 0.26 ± 0.06 and 0.19 ± 0.01, for ETHO and ETHO-DMF_0.5_ respectively.

##### Solvent-Removing Experiments

**Figure 3:**
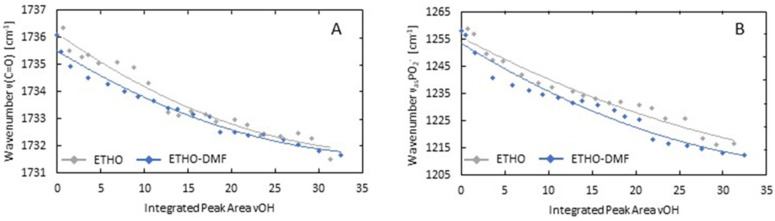
The relationship between the maximum position of νC=O (**A**) and ν_as_PO_2_-band (**B**) and the integrated peak area of νOH band for ETHO (gray line) and ETHO-DMF_0.5_ (blue line) samples.

The effect of the presence of DMF molecules on H-bond (hydrogen-bond) formation between PC and solvent (water/ethanol) molecules is shown in Figure 3.

In the solvent-removing experiments, the solvent molecules were slowly evaporated from both ETHO and ETHO-DMF_0.5_ by incubation at 40 °C. The changes in the amount of solvent were monitored by changes in the integrated peak area of νOH (stretching vibrations of OH groups of solvent molecules) band. The alterations in the number of H-bonds formed between C=O and PO_2_^−^ lipid groups and OH groups of solvent molecules were monitored by changes in the maximum position of νC=O and ν_as_PO_2_^−^ bands, respectively. A brief interpretation of these bands according to [37,38,39] is the following: the band at 1736 cm^−^^1^ is the contribution of stretching vibrations of lipid C=O groups; the band at 1255 cm^−^^1^ is attributed to asymmetric stretching vibrations of lipid PO_2_^−^ groups. The maximum of both bands shifts to the low-wavenumber range with an increase in the number of H-bonds formed between the above-mentioned lipid groups and the OH groups of solvent molecules. As Figure 3A shows, the blue line, which represents the relationship between the maximum position of νC=O band and the integrated peak area of νOH band in ETHO-DMF_0.5_, is shifted to lower wavenumbers in comparison to the gray line which is attributed to ETHO. This indicates that the incorporation of DMF molecules into ETHO induces an increase in the number of H-bonds formed between lipid C=O groups, located in the interfacial region of PC membranes, and the OH groups of solvent molecules. A similar situation was observed for the polar headgroup region of lipid membranes. In Figure 3B the low-wavenumber shift of the blue line compared to the gray line indicates that the presence of DMF molecules induces a rise in the number of H-bonds formed between lipid PO_2_^−^ groups, located in the headgroup region of PC membranes, and the OH groups of solvent molecules. A similar effect of DMF molecules on the headgroup region of lipid membranes was observed for egg PC liposomes [40]. In this study, a DMF-induced increase in hydration level of membrane headgroup region was manifested by the low-wavenumber shift of νPO_2_^−^ band, because of an increase in the number of H-bonds formed between lipid PO_2_^−^ groups and OH groups of water molecules.

##### Temperature-Dependent Studies

The structural changes in pure ETHO and ETHO-DMF_0.5_ triggered by an increase in temperature were studied using FTIR spectroscopy supported by Principal Component Analysis (PCA). PCA calculations were conducted to improve the structural information derived from the spectra of measured samples modulated by temperature. As reported in Figure S1A (Supplementary Materials), a positive loading peak assigned to νOH vibrations, with a maximum centered at lower-wavenumber range, indicates the formation a larger alcohol clusters in ETHO and ETHO-DMF_0.5_ under lower temperatures. These clusters reorganize into smaller ones under the influence of temperature increase, resulting in a shift of νOH band to higher wavenumbers [41].

### 2.3. DMF Entrapment Capacity (EC)

The EC of DMF in ETHO-DMF_0.5_ was evaluated separating the lipid vesicular phase from the aqueous one by ultrafiltration. The EC value, obtained by HPLC after disaggregation of the lipid vesicles, was 91.42 ± 2.5%, suggesting an almost total association of the drug within the PC vesicles, in agreement with FTIR results.

### 2.4. Preparation and Characterization of ETHO Gel

**Figure 4:**
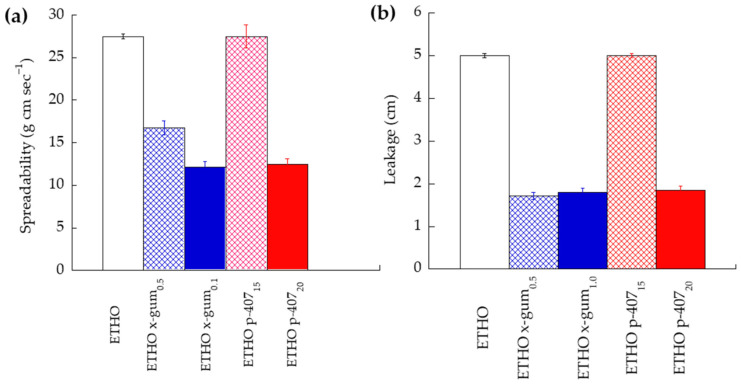
Spreadability (**a**) and leakage (**b**) parameters of ETHO and ETHO gels. Data are the mean of three independent experiments ± s.d.

ETHO were thickened by x-gum (0.5, 1% *w*/*w*) or p-407 (15, 20% *w*/*w*) to obtain semisolid forms suitable for topical application (Table 3). The resulting ETHO gels appeared whitish and homogeneous. The spreadability and leakage of ETHO gels were investigated in vitro to select the more suitable gel for mucosal, ocular, or cutaneous administration. Indeed, the spreadability affects the covering of mucosa (such as the oral one) and lips, or skin area, as well as the extrudability from the container. Moreover, since the spreadability can also influence the gel dosage transfer, it indirectly impacts the therapeutic efficacy [42]. On the other hand, to achieve a sustained effect, the ETHO gels should remain as long as possible on the application site, with minimal leakage. At this regard, the formulation running distance over the vertical plane reflects the leakage. Table 3 and Figure 4 compare spreadability and leakage parameters of ETHO gels and ETHO, taken as control.

The highest spreadability and leakage values were found in the case of ETHO and ETHO p-407_15_, suggesting that p-407 15% *w*/*w* scarcely affected the ETHO liquid consistency. Conversely, p-407 20% *w*/*w* strongly reduced the ETHO spreadability and leakage values. The spreadability and leakage values found in the case of ETHO x-gum_1.0_ were almost superposable to those obtained by ETHO p-407_20_. The halving of x-gum to 0.5% *w*/*w* scarcely affected leakage, while it increased spreadability value. Therefore, ETHO x-gum_0.5_ (hereafter named EG) was selected, being characterized by an intermediate spreadability with respect to the other formulations, and a lower leakage, maintaining its position on the slide also 1 h after placement. The dispersion of x-gum 0.5% *w*/*w* into ETHO-DMF_0.5_ resulted in the formation of an ETHO gel (EG-DMF_0.5_) with the same technological characteristics of the corresponding unloaded one. Table 4 reports acronyms and compositions of the gel formulations employed for further studies.

### 2.5. In Vitro Release Test (IVRT)

**Figure 5:**
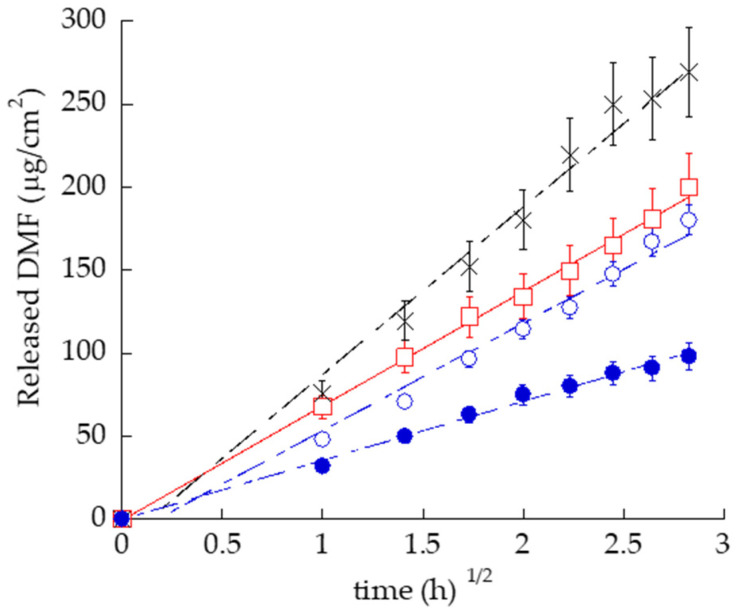
DMF release kinetics from ETHO-DMF_0.5_ (open blue circles), EG-DMF_0.5_ (closed blue circles), G-DMF_0.5_ (red squares), and SOL-DMF_0.5_ (black crosses), as determined by Franz cell associated to PTFE membranes. Data are the mean of six independent experiments ± s.d.

Franz cells associated to synthetic membranes constituted of PTFE were employed to compare the DMF release kinetics from ETHO-DMF_0.5_, EG-DMF_0.5_, G-DMF_0.5_, and SOL-DMF_0.5_, a 0.5 mg/mL DMF solution in ethanol:water 30:70, *v/v*. The PTFE porous synthetic membrane was assembled between the upper and lower compartment of the Franz cell to act as a physical support, to prevent the mixing of donor and receptor phases. As shown in Figure 5, DMF release kinetics followed the order SOL-DMF_0.5_ > G-DMF_0.5_ > ETHO-DMF_0.5_ > EG-DMF_0.5_.

The release rates of DMF, reported in Table 5, were 1.5-fold slower in the case of DMF loaded in ETHO with respect to the drug in solution. As expected, EG-DMF_0.5_ enabled to control drug release, indeed the release rate of DMF was 2.8- and 1.82-fold slower with respect to SOL-DMF_0.5_, and ETHO-DMF_0.5_ respectively. All the differences between R_DMF_ values were statistically significant (*p* < 0.005), apart from the difference between ETHO-DMF_0.5_ and G-DMF_0.5_.

Several plots (zero order plot, first order plot, Higuchi plot and Peppas plots) were drawn in order to understand the DMF release mechanism from ETHO-DMF_0.5_, EG-DMF_0.5_, and G-DMF_0.5_. Equations are reported in S2 in Supplementary Materials, data are shown in Figure S2 and Table 6.

From the results, considering the R^2^ values, in all cases the drug release followed Higuchi order kinetics. The fitting into Korsmeyer–Peppas equation revealed a Fickian diffusion in the case of G-DMF_0.5_ (n = 0.5) and a non-Fickian diffusion mechanism in the case of ETHO-DMF_0.5_ and EG-DMF_0.5_, (0.5 < n < 1) [43].

### 2.6. In Vitro Permeation Test (IVPT)

**Figure 6:**
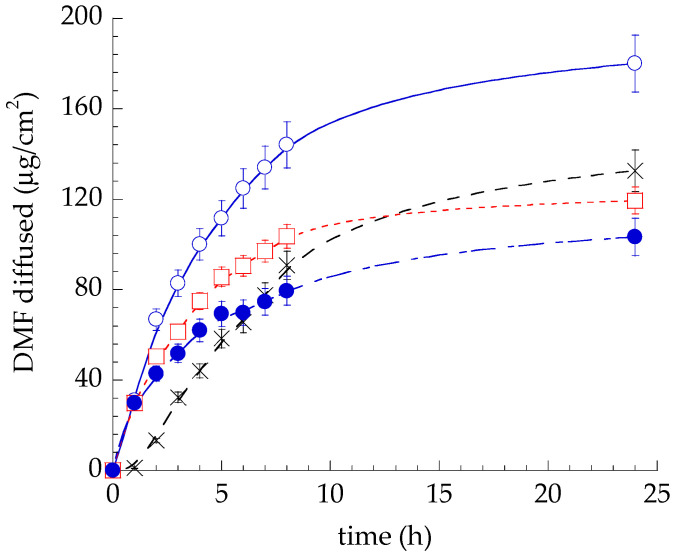
DMF permeability kinetics from ETHO-DMF_0.5_ (blue open circles), EG-DMF_0.5_ (blue closed circles), SOL-DMF_0.5_ (black crosses), and G-DMF_0.5_ (red open squares), as determined by Franz cell associated to STRAT-M^®^ membrane. Data are the mean of six independent experiments ± s.d.

The permeability of DMF loaded in EG-DMF_0.5_ was evaluated and compared to ETHO-DMF_0.5_, and SOL-DMF_0.5_, using Franz cell associated to Strat-M^®^, a synthetic polymeric multimembrane system able to mimic the skin. Strat-M^®^ is made of two polyether sulfone layers overlapped to one polyolefin bottom layer, conferring to the membrane system a skin-like tortuous porous structure [44]. The impregnation with synthetic lipids further imparts to this membrane a skin affinity, recreating hydrophilic and lipophilic compartments, that lend barrier properties. As reported in Figure 6, during the first hour, the DMF profile through ETHO-DMF_0.5_, G-DMF_0.5_, and EG-DMF_0.5_ were superposable, afterwards the fastest kinetic was found in the case of ETHO-DMF_0.5_, followed by SOL-DMF_0.5_, G-DMF_0.5_, and EG-DMF_0.5_. The DMF permeability profile in the case of SOL-DMF was characterized by a Tlag, absent in the case of ETHO-DMF_0.5_, G-DMF_0.5_, and EG-DMF_0.5_. DMF permeation was typically faster within the first 8 h, afterwards it got slower, reaching a plateau at 24 h, as previously found in a study evaluating the ketoprofen permeation from a semisolid dosage form by Franz cells associated to Strat-M membrane [45].

Jss values were calculated from the linear part of the diffusion profiles (1–5 h). ETHO-DMF_0.5_ displayed the highest Kp, as reported in Table 7.

All the differences between Kp values were statistically significant (*p* < 0.005), apart from the difference between ETHO-DMF_0.5_ and SOL-DMF_0.5_.

### 2.7. Citotoxicity Evaluation

**Figure 7:**
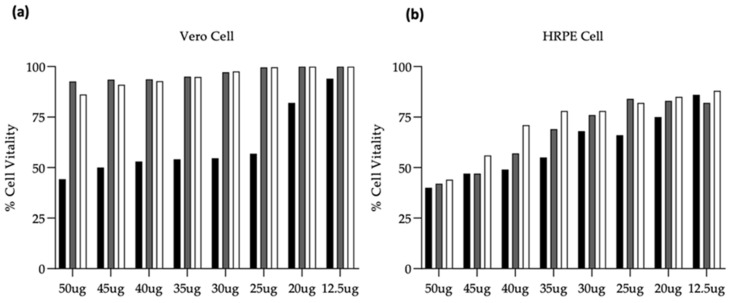
Percentage of cell viability after treatment with SOL-DMF_0.5_ (black), EG-DMF_0.5_ (gray), and ETHO-DMF_0.5_ (white). The neutral red uptake assay provides a quantitative measurement of the number of viable cells measured at OD 540 nm. Panel (**a**) cell viability of the Vero cell line, panel (**b**) cell viability of the HRPE cell line. Data are expressed as mean ± SEM of three different experiments.

DMF concentration and safety of ETHO-DMF_0.5_ and EG-DMF_0.5_ were determined on two different cell lines (Vero and HRPE cells). The cell viability was tested after a 24 h incubation period with the neutral red assay. The neutral red assay determines the accumulation of the neutral red dye in the lysosomes, viable cells can release the incorporated dye in under acidified extracted conditions. In Figure 7 the data show the viability cells at different concentration of SOL-DMF_0.5_, EG-DMF_0.5_, and ETHO-DMF_0.5_. The obtained results demonstrated that (i) the entrapment of DMF in ETHO-DMF_0.5_ and in EG-DMF_0.5_ enabled to reduce its toxicity, (ii) HRPE cells were more susceptible than Vero cells. The concentration of DMF 35 μg/mL was selected for further antiviral activity study, being suitable for both cell lines (70% cell viability).

### 2.8. In Vitro Antiviral Activity

**Figure 8:**
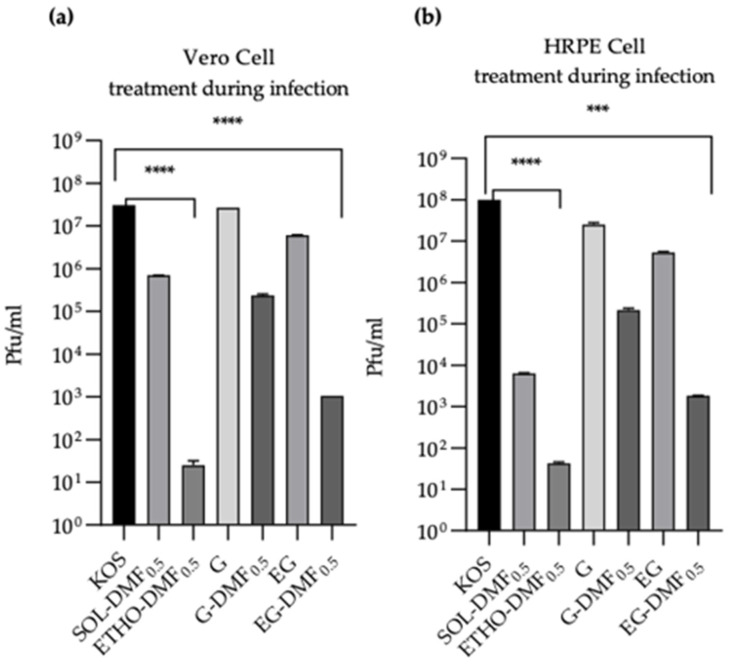
Evaluation of antiviral activity of the treatment during HSV-1 KOS infection on Vero (**a**) or HRPE cells (**b**). Data are expressed as mean ± s.d. of three different experiments: *** *p* values < 0.001, **** *p* values < 0.0001.To test the antiviral activity of the substance after viral entry and during the replication, SOL-DMF_0.5_, ETHO-DMF_0.5_, G-DMF_0.5_, and EG-DMF_0.5_ (DMF 35 μg/mL) were added 1 h or 4 h post-infection (Figure 9). A strong and significant reduction in viral growth was observed in the case of Vero cells treated with ETHO-DMF_0.5_ and EG-DMF_0.5_, with respect to untreated control (Figure 9).

The DMF antiviral activity against HSV-1 was tested by plaque reduction assay in Vero and HRPE monolayer cells. Particularly the antiviral activity was evaluated adding SOL-DMF_0.5_, ETHO-DMF_0.5_, G-DMF_0.5_, and EG-DMF_0.5_ (DMF 35 μg/mL) both simultaneously with the virus at the time of viral absorption, to test the direct action of the formulations on virus, and on cells after viral entry, during the infection. The data in Figure 8 show a significant reduction of plaques when the Vero cells and virus were treated simultaneously with ETHO-DMF_0.5_ and EG-DMF_0.5_, with respect to control infected cells (KOS) (Figure 8a). On the other hand, a significant reduction on viral particles was observed under HRPE cell infection with the virus and simultaneous treatment with SOL-DMF_0.5_, ETHO-DMF_0.5_, and EG-DMF_0.5_, with respect to control infected cells (KOS) (Figure 8b).

**Figure 9:**
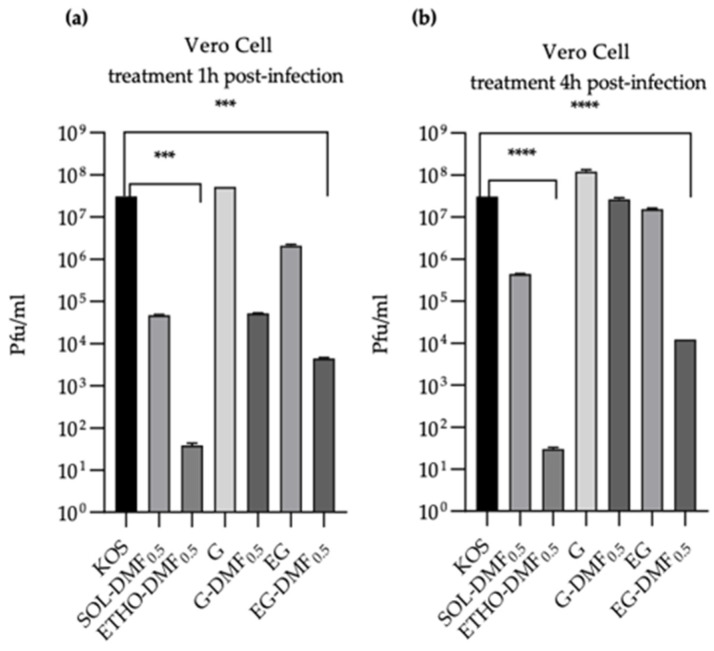
Evaluation of antiviral activity of the treatment post HSV-1 KOS infection on Vero (**a**) or HRPE cells (**b**). Data are expressed as mean ± s.d. of three different experiments: *** *p* values < 0.001, **** *p* values < 0.0001.

**Figure 10:**
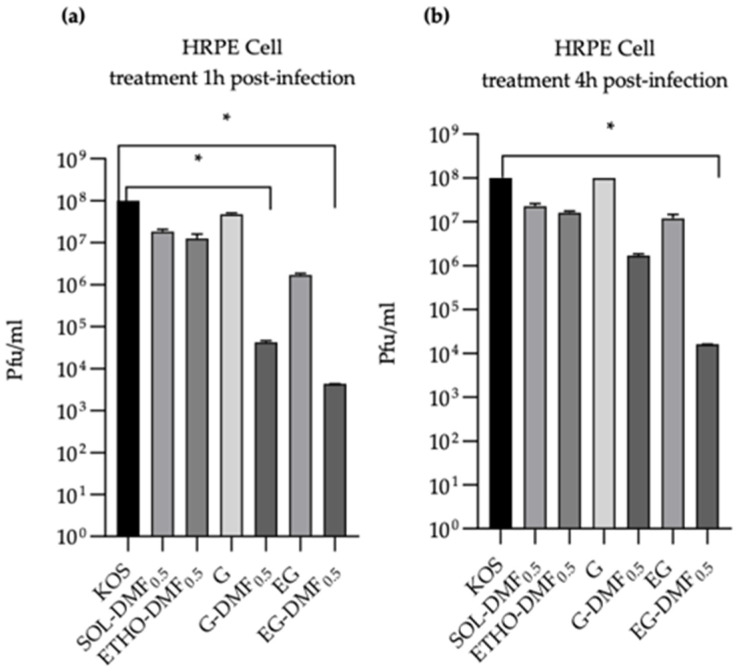
Evaluation of antiviral activity on HRPE cells treated by the indicated formulations 1 h (**a**), or 4 h (**b**) post-infection. Data are expressed as mean ± s.d. of three different experiments: * *p*-values < 0.05.

In the case of HRPE cells, the highest antiviral activity was exerted by EG-DMF_0.5_, both 1 h and 4 h post-infection, with respect to untreated control (Figure 10).

### 2.9. Patch Test

A patch test was performed to evaluate the safeness of EG-DMF_0.5_ for cutaneous application. The gel applied for 48 h under occlusive condition on the healthy skin of 20 volunteers can be classified as “not irritating”, since it resulted in a 0.15 average irritation index, therefore well below the threshold of 0.5.

## 3. Discussion

The results of this study suggested the possibility to employ DMF loaded in an ETHO gel in the topical treatment of HSV-1 infections. The purposes of developing an ETHO formulation with respect to a conventional form were (i) to enhance DMF transdermal delivery and ocular absorption, (ii) to prolong DMF antiviral action by improving its interaction with the skin, lips or eye and (iii) to increase DMF bioavailability, reducing the therapeutic dosage, while minimizing toxic side effects.

The ETHO excipients were chosen based on previous formulative studies investigating the influence of vesicular nanosystem composition on their size distribution, morphology and stability [46,47]. Both DMF 0.5 and 1.0 mg/mL concentrations were considered on the basis of previous investigations about the nanoencapsulation of DMF in solid lipid nanoparticles and in transethosomes [20,48]. Notably, the loading of DMF 0.5 mg/mL in transethosomes, resulted in stable vesicles, suitable for cutaneous administration. Accordingly, in the present formulative study the entrapment of DMF 0.5 mg/mL in ETHO resulted in stable vesicles, with mean diameter compatible for topical administration on skin, lips and eye, and a homogeneous size distribution. ETHO-DMF_0.5_ visualized by cryo-TEM revealed the presence of multilamellar bi-layered vesicles due to the PC self-organization in ethanol/water mixture. The ETHO-DMF_0.5_ structural characterization by FTIR further demonstrated that the presence of DMF changes the occupation of both the interface and the headgroup lipid membrane regions with solvent molecules, possibly stabilizing the vesicle ultrastructure.

The multilamellar organization of ETHO-DMF_0.5_ enabled to sustain DMF release with respect to SOL-DMF_0.5_, as demonstrated by Franz cell experiments performed with PTFE membrane.

On the other hand, the use of STRAT-M^®^, employed to mimic the biological epithelia, revealed that ETHO-DMF_0.5_ improved DMF permeability with respect to SOL-DMF_0.5_, suggesting the capability of ETHO vesicles to enhance DMF diffusion through the membrane.

In order to thicken the ETHO liquid dispersion, both p-407 and x-gum were considered. At 15–25% *w*/*w* concentrations, p-407 in water leads to thermo-reversible gels, passing from a low viscosity solution, to a transparent viscous gel above the transition temperature, suitable for administration on skin and mucosae. Different studies report the use of p-407 as thickeners of nanocarrier systems [49,50,51]. Particularly, in a previous study, in order to thicken an ETHO dispersion for cutaneous administration of caffeic acid, we directly added p-407 15%, *w*/*w* to the dispersion, resulting in an ETHO gel with semisolid consistency, maintaining the typical supramolecular organization of PC [51]. X-gum is widely employed for pharmaceutical applications, typically 0.5–1% *w*/*w* of x-gum can be employed to confer to a disperse system the suitable viscosity for topical administration, as previously demonstrated by our group [20,52,53]. The formulative study here described enabled to select x-gum 0.5%, *w*/*w*, as thickener to confer to ETHO-DMF_0.5_ the appropriate technological features (i.e., spreadability and leakage) for topical administration, prolonging the contact time with the biological surfaces.

As expected, the obtained nanovesicle scaffold EG-DMF_0.5_ enabled to control DMF release with respect to SOL-DMF and ETHO-DMF_0.5_, as demonstrated by Franz cell associated with PTFE membrane. With regard to the mechanism of DMF release, all formulations followed the Higuchi’s square root model, as previously found in the case of vesicular gels [54]. Indeed, the drug release from nano-vesicular gels was reported to depend on the PC bilayer organization, resulting in a core-shell controlled-release delivery system. Considering the diffusional exponent “n”, which is indicative of the transport mechanism described by Korsmeyer–Peppas, the plain G-DMF_0.5_. displayed a Fickian release, suggesting that the DMF release could be governed by diffusion through the gel matrix, as previously observed [45]. On the other hand, the presence of vesicles in ETHO-DMF_0.5_ and EG-DMF_0.5_ resulted in an anomalous non Fickian release, suggesting a superposition of diffusion and relaxation phenomena [43].

Furthermore, the Kp value of DMF through EG-DMF_0.5_, evaluated by STRAT-M^®^, was 1.55-fold lower with respect to ETHO-DMF_0.5_, while the total amount of DMF diffused after 24 h in the case of EG-DMF_0.5_ was 1.72- and 1.28-fold lower with respect to ETHO-DMF_0.5_ and SOL-DMF_0.5_, respectively. This behavior suggests that both the multilamellar PC organization of the vesicles and the presence of x-gum network in the dispersing phase contribute to sustain the diffusion of DMF through EG-DMF_0.5_. STRAT-M^®^, was efficaciously employed as a characterization tool for pre-development stage, nonetheless the transepithelial effect, including skin and mucosa retention studies, will be further evaluated using mucosa and epidermal sheets of murine skin.

The plaque reduction assay, conducted after selecting the safe drug concentration, demonstrated the anti-HSV-1 activity of DMF on Vero and HRPE cells. Particularly, ETHO-DMF_0.5_ and EG-DMF_0.5_ were able to inhibit the viral infection or growth, both at the time of viral absorption, or 1 h and 4 h after the cell infection. Notably, in the case of Vero cells both ETHO-DMF_0.5_ and EG-DMF_0.5_ induced a strong and significant reduction in viral infection and viral growth, while, in the case of HRPE cells the reduction was observed only by EG-DMF_0.5_. Conversely, G-DMF_0.5_ antiviral activity was less effective with respect to the ETHO gel, especially 4 h after the cell infection, suggesting that the entrapment of DMF in ETHO vesicle within the x-gum network can sustain its activity, in agreement with release and diffusion data. It is noteworthy that in the case of HRPE cells the ETHO-DMF_0.5_ dispersion exerted a scarce anti HSV-1 action, while the virucidal activity of its thickened form was almost double (Figure 10), possibly indicating that the presence of x-gum was crucial to promote the DMF contact with the infected cells.

The results agree with many studies demonstrating the ability of nanovesicle forms to enhance dermal and ocular drug delivery [24,25,51,55,56]. For instance, a pilot clinical study demonstrated the improved clinical efficacy of acyclovir-loaded ETHO preparation with respect to a commercial acyclovir cream in the treatment of recurrent herpes labialis [57], while very recently a valacyclovir-loaded liposomal formulation has been developed to treat HSV-1 infections [58].

In the case of ophthalmic applications, nano-vesicular systems offer advantages over other delivery systems in promoting intimate contact with corneal and conjunctival surfaces, thus improving the ocular drug absorption [31,56,59,60,61]. For instance ganciclovir-loaded liposomes proposed in the treatment of ocular infections demonstrated a high drug transcorneal permeability, due to an interaction between liposomes and the corneal epithelial surface [56]. Moreover ketoconazole-loaded transethosomes were able to enhance the drug ocular permeation, promoting the antifungal activity, penetrating deeply into the posterior eye segment, without any toxic effects [60]. Beyond the antiviral efficacy of the EG-DMF_0.5_ formulation developed in the present study, its safeness, demonstrated by patch test assay, suggests its suitability in the treatment of HSV-1 infection, nonetheless further study will be required to confirm the formulation usefulness for ophthalmic administration.

## 4. Materials and Methods

### 4.1. Materials

Dimethyl fumarate (dimethyl (E)-but-2-enedioate, DMF), poloxamer 407 (p-407), xanthan gum (x-gum), blue Coomassie, and deuterated water (D_2_O) were purchased from Merck Life Science S.r.l. (Milan, Italy). The soybean lecithin (PC) (90% phosphatidylcholine) was Epikuron 200 from Lucas Meyer (Hamburg, Germany). Polytetrafluoroethylene (PTFE, Whatman^®^) (pore size 200 nm), and STRAT-M^®^ membranes were purchased from Merck Life Science S.r.l. (Milan, Italy). Solvents were of HPLC grade, and all other chemicals were of analytical grade.

### 4.2. Ethosome Preparation

ETHO preparation was obtained by the cold method [55]. Briefly, PC was solubilized in ethanol (30 mg/mL) under stirring at 750 rpm (IKA RCT basic, IKA^®^-Werke GmbH & Co. KG, Staufen, Germany), after complete solubilization, bi-distilled water was dropwise added to the PC solution up to a final 70:30 (*v/v*) water/ethanol ratio. The magnetic stirring was maintained for 30 min. To load DMF, the drug (0.5 or 1 mg/mL) was solubilized in the PC ethanol solution before adding water. ETHO for FTIR experiments were prepared by the same protocol, employing D_2_O instead of H_2_O.

### 4.3. Photon Correlation Spectroscopy

Vesicle size distribution was measured using a Zetasizer Nano-S90 (Malvern Instr., Malvern, UK) with a 5 mW helium neon laser and a wavelength output of 633 nm. Measurements were performed at 25 °C at a 90° angle and a run time of at least 180 s. Samples were diluted with bi-distilled water in a 1:10 *v*/*v* ratio. Data were analyzed using the “CONTIN” method [62]. Measurements were performed thrice for 3 months after ETHO production, on ETHO stored at 22 °C, calculating the mean ± standard deviation (s.d.). The SIR of vesicles was expressed calculating the difference between Z Average mean diameter of ETHO stored for 3 months and Z Average mean diameter of ETHO measured the day after preparation, as follows:

(1) $$\mathrm{SIR}=\frac{{Z\;\mathrm{Average}}_{\mathrm{day}\;90}-{Z\;\mathrm{Average}}_{\mathrm{day}\;1}}{{Z\;\mathrm{Average}}_{\mathrm{day}\;1}}\times 100$$

The statistical differences were evaluated by *t* student test, GraphPad Prism 9 software (GraphPad Software Inc., San Diego, CA, USA), considering values of *p* < 0.05 as statistically significant.

### 4.4. Cryo-Transmission Electron Microscopy

For cryo-TEM analyses, samples were vitrified following a method previously reported [51]. Namely, a 2 μL aliquot of sample was put for few seconds on a lacey carbon filmed copper grid (Science Services, München, Germany). After removing most of the liquid by a blotting paper, a thin film stretched over the lace holes was obtained. Vitrification was achieved by rapid immersion of specimen into liquid ethane cooled to approximately 90 K (−180 °C) by liquid nitrogen in a temperature-controlled freezing unit (Leica EMGP, Leica, Germany). The sample preparation procedure was conducted at controlled constant temperature in the Leica EMGP chamber. The vitrified specimen was transferred to a Zeiss/Leo EM922 Omega EFTEM (Zeiss Microscopy GmbH, Jena, Germany) transmission electron microscope using a cryoholder (CT3500, Gatan, Munich, Germany). During the microscopy observations, sample temperature was kept below 100 K. Specimens were examined with reduced doses ≈1000–2000 e/nm^2^ at 200 kV. Zero-loss filtered images (∆E = 0 eV) were recorded by a CCD digital camera (Ultrascan 1000, Gatan, Munich, Germany) and analyzed by a GMS 1.9 software (Gatan, Munich, Germany).

### 4.5. Structural Characterization of ETHO by FTIR

In temperature-dependent studies for ETHO and ETHO-DMF_0.5_ samples, FTIR spectra were collected on a Nicolet Avatar FTIR spectrometer (GMI, Ramsey, MN, USA). The 32 scans were collected for each spectrum at a resolution of 2 cm^−^^1^. During a heating cycle, CaF_2_ windows and a 56-µm spacer to ensure a constant sample thickness were used. Spectra were measured in temperature range from 10 to 80 °C, with intervals of 5 °C. The samples were allowed to equilibrate for 5 min prior to the acquisition of each spectrum. High Stability Automatic Temperature Controller P/N 20120 series (Specac Ltd., Orpington, UK) as an external heating system was used for FTIR measurements.

For solvent-removing experiments samples was incubated at 40 °C in order to induce a slow evaporation of solvent molecules from ethosomes dispersions. ATR accessory (PIKE, Madison, WI, USA) with a ZnSe crystal with 10 reflections and a face angle of 45° was equipped additionally to the Nicolet Avatar FTIR spectrometer. An F25 Julabo water bath (Julabo, Labrotechnic, GMbH) was used as an external heating system.

Prior to performing PCA calculations, the pretreatment steps of FTIR spectra of ethosomes were as follows: (1) the subtraction of the solvent spectrum from the spectrum of the sample under study; (2) noise reduction using the Savitzky–Golay fuction with the smoothing filter was with 17-point window and a polynomial of order 2; (3) a baseline correction process with a linear function; and (4) application of standard normal variate (SNV) normalization and mean center processes to the analytical data. These spectral pretreatments and PCA calculations were accomplished using version 8.0 of the GRAMS/32 AI software (Galactic Industries Corporation, Thermo Scientific, Warsaw, Poland) and version 6.1 of the PLS Toolbox (Eigenvector Research, ICN, Wenatchee, WA, USA) for the Matlab R2009a software (The MathWorks Inc., Natick, MA, USA).

### 4.6. Evaluation of DMF EC in Ethosome

The EC of DMF in ETHO-DMF_0.5_ was determined by ultrafiltration, 24 h after preparation using a centrifugal filter device (Microcon centrifugal filter unit YM-10 membrane, NMWCO 10 kDa, Sigma-Aldrich, St. Louis, MO, USA) and HPLC analysis as below reported. Namely, 500 μL of DMF-loaded ETHO were poured in the sample reservoir part of the device and subjected to ultrafiltration (Spectrafuge™ 24D Digital Microcentrifuge, Woodbridge, NJ, USA) at 4000 rpm for 15 min. Afterwards, both retentate and filtrate fractions were withdrawn respectively from the sample reservoir part or the vial, and diluted with ethanol (1:10, *v/v*). Before HPLC analysis, the diluted retentate was stirred for 30 min and filtered by nylon syringe membranes (0.22 μm pore diameter), while the filtrate fraction was analyzed as such. The EC was determined as follows:

EC = DMF/T_DMF_ × 100 (2)

where DMF is the amount of drug measured by HPLC and T_DMF_ is the total amount of DMF employed for ETHO preparation.

### 4.7. Preparation and Characterization of Ethosomal Gels

To prepare a viscous gel, alternatively x-gum or p-407 were employed. X-gum (0.5 or 1%, *w*/*w*) was added to ETHO under magnetic stirring for 30 min, up to complete dispersion. P-407 (15 or 20%, *w*/*w*) was gradually added to ETHO at 4 °C in an ice bath DMF under magnetic stirring, up to complete dispersion. The resulting ETHO gels (ETHO x-gum_0.5_, ETHO x-gum_1.0_, ETHO p-407_15_ and ETHO p-407_20_) were tested for spreadability and leakage.

#### 4.7.1. Spreadability Studies

The spreading capacity of gels (ETHO x-gum_0.5_, ETHO x-gum_1.0_, ETHO p-407_15_ and ETHO p-407_20_) was evaluated at ambient temperature (25 °C), 24 h after gel preparation [42]. Precisely, 150 mg of gel were placed in the center of a Petri dish (3 cm diameter) and then subjected to pressure by a glass dish carrying a 50 g mass. The diameter of the area occupied by the formulation in a predetermined time (10 s) was measured. The spreadability test was performed three times and the mean values ± standard deviations were calculated using the following equation:

S = m × l/t(3)

where S is the spreadability of the gel formulation, m is the weight (g) tied on the upper plate, t is the time (10 s), and l is the diameter (cm) of the area occupied by the gel in 10 s under pressure [42].

#### 4.7.2. Leakage Test

To test leakage and adhesion of gels (ETHO x-gum_0.5_, ETHO x-gum_1.0_, ETHO p-407_15_ and ETHO p-407_20_), phosphate buffer pH 7.4 was prepared, afterwards agar (1.5% *w*/*w*) was added and stirred at 95 °C until solubilization. The gels obtained after cooling were then cut to obtain rectangular agar slides. The gels were colored for the leakage test by dissolving blue coomassie (0.05% *w*/*w*), afterwards, 50 mg of colored formulations were placed onto one end of agar slide placed in a Petri plate. The Petri plate was vertically put at an angle of 90° on one of the inner walls of a transparent box, maintained at 37 °C ± 1 °C. The running distance along the slide was measured 10 s after the gel placement. Gel leakage, expressed as percentage of the difference between the total length of the agar slide and the running distance of the gel, was measured three times, and the mean values ± standard deviations were calculated.

### 4.8. Franz Cell Diffusion Experiments

Franz cells (orifice diameter 0.9 cm; PermeGear Inc., Hellertown, PA, USA) were employed for IVRT and for IVPT. Notably PTFE membranes were used for IVRT, while STRAT-M^®^ membranes were employed for IVPT [20]. Both for IVRT and IVPT, samples of dried membranes were rehydrated by immersion in ethanol/water 50:50, *v*/*v* for 1 h, before assembling in Franz-type diffusion cells. The receptor compartment of the cell contained 5 mL of ethanol:water 50:50, *v*/*v* in order to assure sink conditions [63], stirred at 500 rpm by a magnetic bar and thermostated at 32 ± 1 °C during the experiments [64]. Five hundred microliters of DMF-loaded ETHO (ETHO-DMF_0.5_), DMF ethanolic solution (ethanol:water 30:70, *v/v*) (DMF 0.5 mg/mL) (SOL-DMF_0.5_), or ETHO gel (EG-DMF_0.5_), were placed on the membrane surface in the donor compartment that was afterwards sealed to avoid evaporation. At predetermined time intervals comprised between 1 and 24 h, samples (0.5 mL) of receptor phase were withdrawn and analyzed by HPLC to evaluate the DMF content. Each removed sample was replaced with an equal volume of simple receptor phase. The DMF concentrations were determined six times in independent experiments, the mean values ± s.d. were calculated.

#### 4.8.1. In Vitro Release Test (IVRT)

For data analysis, in the case of IVRT, DMF amount (μg/cm^2^) was plotted as a function of the square root of time [63]. To compare the DMF release kinetics from the different dispersions, the following parameters were evaluated: “R_DMF_” the slope of the cumulative amount of DMF released versus the square root of time; lag-time “T_lag_” extrapolated from the intercept of the release profile with *x*-axis; and “A_DMF_” the cumulative amount of DMF released at the last sampling time (8 h). The release kinetics parameters were evaluated by mathematical models (KinetDS, Aleksander Mendyk) [43], as reported in S2. Both R^2^ and n values were taken as reference, considering 0.5 as index of Fickian diffusion model, and 0.5 < n < 1.0 of non Fickian one.

#### 4.8.2. In Vitro Skin Permeation Test (IVPT)

In the case of IVPT, to analyze data, Fick’s law was considered since it describes the steady-state permeation through the skin, assuming that, under sink conditions, drug concentration in the receptor compartment is negligible with respect to that in the donor compartment [39]. The steady-state flux of drug per unit area corresponds to the slope of the linear part of the curves “Jss” (µg/cm^2^/h) [65]. DMF permeability coefficients “Kp” values were calculated considering the steady-state portion of DMF cumulative penetration profiles versus time. Kp was calculated according to Equation (4):

Kp = Jss/Cd(4)

where Cd is the drug concentration in the donor compartment.

### 4.9. HPLC Analysis

HPLC analyses were performed using Perkin Elmer, Series 200 HPLC Systems equipped with a micro-pump, an auto sampler, and an UV-detector operating at 216 nm. A stainless-steel C-18 reverse-phase column (15 × 0.46 cm) packed with 5 μm particles (Hypersil BDS C18 Thermo Fisher Scientific S.p.A., Milan, Italy) was eluted at a flow rate of 1 mL/min with a mobile phase containing acetonitrile/water 40:60 *v/v*.

### 4.10. Antiviral Activity Study against HSV-1

#### 4.10.1. Cell Culture

Vero and HRPE were cultivated in Eagle’s minimum essential medium (DMEM), or DMEM-F12 supplemented with 10% fetal bovine serum (FBS), 100 mg/mL penicillin and 100 mg/mL streptomycin, incubated at 37 °C under 5% CO_2_ in an incubator. Cells were seed at 5 × 10^5^ per well in a six- well plate, 24 h prior to plaque assay [66].

#### 4.10.2. Cytotoxicity

Cell viability was determined by neutral red uptake assay. The cells were seeded in triplicate in a 96-well plate at a density of 20 × 10^3^ in 100 mL of DMEM high glucose and 10% FCS medium for Vero cell line and DMEM-F12 with 10% FBS for HRPE cells. The next day the cells were treated with different concentrations (12.5, 20, 25, 30, 35, 40, 45, 50 μg/μL) of DMF solution or ETHO-DMF_0.5_ or EG-DMF_0.5_. After 24 h the medium was removed from the 96-well plates, the cells were gently rinsed with phosphate buffered saline (PBS), 250 μL neutral red (NR) dye medium was added to the wells (25 μg/mL NR concentration), and the plates were incubated (37 ± 1 °C, 90 ± 5% humidity, and 5.0 ± 1% CO_2_/air) for three hours. After incubation, the NR medium was removed, the cells were rinsed with PBS, and 150 μL of a desorbed solution (50% bi-distilled water, 49% ethanol, 1% acetic acid) was applied. The plates were shaken on a microtiter plate shaker for 45 min to extract NR from the cells and form a homogeneous solution. The absorption (i.e., OD measurement) of the resulting-colored solution was measured (within 60 min of adding the desorb solution) at 540 nm in a spectrophotometric microtiter plate reader.

#### 4.10.3. Herpes Virus Stock Generation

Vero cells 2 × 10^7^ in 10–20 mL of cell culture medium were seeded into a 175 cm^2^ tissue culture flask and incubate overnight at 37 °C in a humidified 95% air-5% CO_2_ incubator. Vero cells were infected with herpes simplex virus strain KOS, at a multiplicity of infection (M.O.I) of 0.01. The cells were incubated for 1 h at 35 °C to allow adsorption of the virus to the cells. The flasks were rocked every 15 min to evenly distribute the inoculum. The virus inoculum was aspirated, and cell culture medium was added up to a final volume of 10–20 mL per flask. Infected cells were incubated at 35 °C in a humidified 95% air-5% CO_2_ incubator for 36–48 h, until complete cytopathic effect (CPE) was reached. Cells and supernatant were collected, cells were removed by low-speed centrifugation, and the supernatant was centrifugated a 20,000 min^−1^ for 30 min. The obtained pellet was resuspended in 1 mL of medium, aliquoted, and kept at −80 °C until use [66].

#### 4.10.4. Titration of Virus by Plaque Assay

The viral preparation was titrated on Vero cells. One day prior to titration, 6-well tissue culture plates with 0.5 × 10^6^ Vero cells per well were prepared. The virus was thawed on ice and sonicated for a few seconds prior to infection, to separate virus particles. A series of ten-fold dilutions (10^−^^2^–10^−^^10^) of the virus stock in 1 mL cell culture medium without serum in Eppendorf tubes were prepared and added to each well containing cells. The cells were incubated for 1 h at 35 °C, to allow adsorption of the virus to the cells. After 1 h of infection, the viral inoculum was removed, and the monolayer was overlayed with 3 mL of 1% methylcellulose (Sigma-Aldrich). The plates were incubated for 3–4 days until well-defined plaques were visible. The methylcellulose medium was removed from the wells and stained for 10–20 min with 2 mL of crystal violet staining solution to fix the cells and the virus. The number of plaques was counted, the average for each dilution (n = 3) was determined and multiplied by 10 to the power of the dilution to obtain the number of plaque forming units per mL (PFU/mL) [66].

#### 4.10.5. Antiviral Activity Assay

The inhibition of virus replication was measured by plaque assay. HSV-1 strain KOS 1 × 10^5^ pfu/mL and cells were incubated with SOL-DMF_0.5_, ETHO-DMF_0.5_, G-DMF_0.5_, and EG-DMF_0.5_ at the time of infection. Alternatively, the same forms were added 1 h and 4 h after cell infection. The positive control were cells infected with the virus and medium. Twenty-four h post-infection the medium and cells of each sample were collected and titrated. After 1 h at 35 °C to allow viral adsorption, the plates were washed and the medium replaced with 3 mL of 1% methylcellulose, to prevent the formation of secondary plaques, and incubated for 3–4 days at 35 °C until the appearance of lysis plaques. Afterwards cells were fixed and stained with a 1% solution of crystal violet to determine the number of plaques [66]. The antiviral activity was evaluated as plaque reduction with respect to control cells.

### 4.11. Statistical Analysis

All experiments were repeated 3–6 times and statistical values were expressed as the mean ± standard deviation (SD). For all data analysis, GraphPad Prism 9 software (GraphPad Software Inc., San Diego, CA, USA) was used. Values of *p* < 0.05 were considered statistically significant.

### 4.12. Patch Test

An in vivo irritation test was performed to evaluate the effect of EG-DMF_0.5_ applied in single dose on the intact human skin. The occlusive patch test was conducted at the Cosmetology Center of the University of Ferrara, following the basic criteria of the protocols for the skin compatibility testing of potentially cutaneous irritant cosmetic ingredients on human volunteers (SCCNFP/0245/99). The protocol was approved by the Ethics Committee of the University of Ferrara, Italy (study number: 170583). The test was run on 20 healthy volunteers of both sexes, which gave a written consent to the experimentation. Subjects affected by dermatitis; with history of allergic skin reaction or under anti-inflammatory drug therapy (either steroidal or non-steroidal) were excluded. Ten milligrams of EG-DMF_0.5_, were posed into aluminum Finn chambers (Bracco, Milan, Italy), and applied onto the skin of the forearm or the back protected by self–sticking tape. Particularly samples were directly applied into the Finn chamber by an insulin syringe, left in contact with the skin surface for 48 h. Skin irritative reactions (erythematous and/or edematous) were evaluated 15 min and 24 h after removing the patch and cleaning the skin from sample residual. Erythematous reactions have been sorted out into three groups, according to the reaction degree: light, clearly visible and moderate/serious erythema. The average irritation index was calculated as the sum of erythema and edema scores and expressed according to a scale considering 0.5 as the threshold above which the product is to be classified as slightly irritating, 2.5–5 as moderately irritating and 5–8 as highly irritating.

## Figures and Tables

**Table 1:** Composition of ethosomes.

Formulation	PC ^1^ % *w*/*w*	Ethanol % *w*/*w*	DMF ^2^ % *w*/*w*	Water % *w*/*w*
ETHO	0.90	29.10	-	70.00
ETHO_-_DMF_0.5_	0.90	29.05	0.05	70.00
ETHO-DMF_1.0_	0.90	29.00	0.10	70.00

^1^: soy phosphatidylcholine; ^2^: dimethylfumarate.

**Table 2:** Size distribution parameters of ethosomes, as determined by PCS.

Formulation	Z Average (nm)	Typical Intensity Distribution (nm)	Dispersity Index	SIR *
ETHO	212.25 ± 15.13	237.1 (100%)	0.15 ± 0.07	11.9 ± 0.9
ETHO_-_DMF_0.5_	223.22 ± 13.60	234.1 (100%)	0.12 ± 0.05	8.88 ± 0.5
ETHO-DMF_1.0_	231.00 ± 10.81	256.5 (94.2%) 4158 (5.8%)	0.22 ± 0.01	16.01 ± 1.2

* Size Increase Ratio, as reported in Equation (1).

**Table 3:** Composition, spreadability and leakage parameters of the indicated formulations.

Formulation	PC ^1^ % *w*/*w*	Ethanol % *w*/*w*	Water % *w*/*w*	Thickener % *w*/*w*		Spreadability ^4^ (g·cm/s)	Leakage ^5^ (cm)
				x-gum ^2^	p-407 ^3^		
ETHO	0.90	29.10	70.00	-	-	27.50 ± 0.25	5.00 ± 0.04
ETHO x-gum_0.5_	0.90	29.10	69.50	0.5	-	16.75 ± 3.40	1.67 ± 0.40
ETHO x-gum_1.0_	0.90	29.10	69.00	1.0	-	12.16 ± 3.00	1.72 ± 0.26
ETHO p-407_15_	0.90	29.10	55.00	-	15.0	27.50 ± 5.55	5.00 ± 0.00
ETHO p-407_20_	0.90	29.10	50.00	-	20.0	12.50 ± 3.15	1.85 ± 0.61

^1^: soy phosphatidylcholine; ^2^: xanthan gum; ^3^: poloxamer 407; ^4^: calculated as reported in Equation (3); ^5^: running distance along the slide; data are the mean of three independent measurements ± s.d.

**Table 4:** Composition of the selected gel formulations.

Formulation	PC ^1^ % *w*/*w*	Ethanol % *w*/*w*	Water % *w*/*w*	DMF ^2^ % *w*/*w*	x-gum % *w*/*w*
EG-DMF_0.5_	0.90	29.05	69.50	0.05	0.50
G-DMF_0.5_	-	-	99.45	0.05	0.50
EG	0.90	29.10	69.50	-	0.50
G	-	-	99.50	-	0.50

^1^: soy phosphatidylcholine; ^2^: dimethylfumarate.

**Table 5:** IVRT parameters of the indicated forms, as determined by Franz cell associated to PTFE membranes.

IVRT Parameters	ETHO_-_DMF_0.5_	SOL-DMF_0.5_	EG-DMF_0.5_	G-DMF_0.5_
R_DMF_^1^ ± s.d. (μg/cm^2^/h)	64.60 ± 5.40	100.66 ± 18.12	35.44 ± 3.15	68.85± 7.35
T_lag_ ^2^ ± s.d. (h)	0.18 ± 0.01	0.15 ± 0.01	0.00 ± 0.01	0.00 ± 0.01
A_DMF_ ^3^ ± s.d. (μg/cm^2^)	180.00 ± 18.21	269.33 ± 42.02	98.00 ± 2.02	200 ± 20.2

^1^: DMF release rate; ^2^: lag-time; ^3^: amount of DMF released after 8 h; DMF was always 0.5 mg/mL; data are the mean of six independent Franz cell experiments ± s.d.

**Table 6:** Kinetic data of the indicated formulations.

Formulation	Zero Order Plot (R^2^)	First Order Plot (R^2^)	Higuchi Plot (R^2^)	Peppas Plot (n)
ETHO-DMF_0.5_	0.9836	0.9827	0.9841	0.6408
EG-DMF_0.5_	0.9578	0.9096	0.9944	0.5311
G-DMF_0.5_	0.9638	0.9481	0.9974	0.5027

**Table 7:** IVPT parameters of the indicated forms, as determined by Franz cell and STRAT-M^®^.

IVPT Parameters	ETHO-DMF_0.5_	SOL-DMF_0.5_	EG-DMF_0.5_	G-DMF_0.5_
Jss ^1^ (mg cm^−2^ h^−1^)	15.16 ± 1.52	14.71 ± 0.40	9.77 ± 1.81	13.56 ± 2.84
T_lag_^2^ ± s.d. (h)	0.00 ± 0.01	0.55 ± 0.02	0.00 ± 0.01	0.00 ± 0.01
Kp ^3^ (cm h^−1^ 10^−3^)	30.32 ± 3.04	29.42 ± 0.8	19.54 ± 3.62	27.12 ± 5.68
A_DMF_^4^ (μg cm^−2^)	178.10 ± 10.52	132.6 ± 9.2	103.33 ± 4.15	118.80 ± 6.22

^1^: steady-state flux per unit area, ^2^: lag-time; ^3^: permeability coefficient; ^4^: cumulative amount of DMF diffused at 24 h; data are the mean of six independent Franz cell experiments ± s.d.

## Data Availability

The data presented in this study are available on request from the corresponding author. The data are not publicly available due to privacy restrictions.

## Supplementary Materials

The supporting information can be downloaded at: https://www.mdpi.com/article/10.3390/ijms24044133/s1.
