# Supplementary material for: Ethosomal Gel for Topical Administration of Dimethyl Fumarate in the Treatment of HSV-1 Infections
Source: Int J Mol Sci. 2023 Feb 18;24(4):4133. doi: 10.3390/ijms24044133 (PMC9967198; doi:10.3390/ijms24044133)
Supplement: Supplementary file 1 [file ijms-24-04133-s001.zip › ijms-2211458-SI.pdf]

Ethosomal Gel for Topical Administration of Dimethyl Fumarate in the Treatment of HSV-1 Infections

Mariaconcetta Sicurella [1], Walter Pula [2], Karolina Musiał [3], Katarzyna Cieślik-Boczula [3], Maddalena Sguizzato [2], Agnese Bondi [2], Markus Drechsler [4], Leda Montesi [5], Elisabetta Esposito [2,*] and Peggy Marconi [2]

S1. FTIR Temperature-dependent studies

The structural changes in pure ETHO and ETHO-$DMF_{0.5}$ triggered by an increase in temperature were studied using FTIR spectroscopy supported by Principal Component Analysis (PCA). PCA calculations were conducted in order to improve the structural information derived from the spectra of measured samples modulated by temperature. For each sample, the PCA model was separately performed for three different vibrational regions: 4000 – 3000 cm^{-1} region assigned to the νOH bands; 3055 – 2780 cm^{-1} region assigned to the νCH bands, and 1770 – 1705 cm^{-1} region assigned to the νC=O bands, as reported in Figure S1.

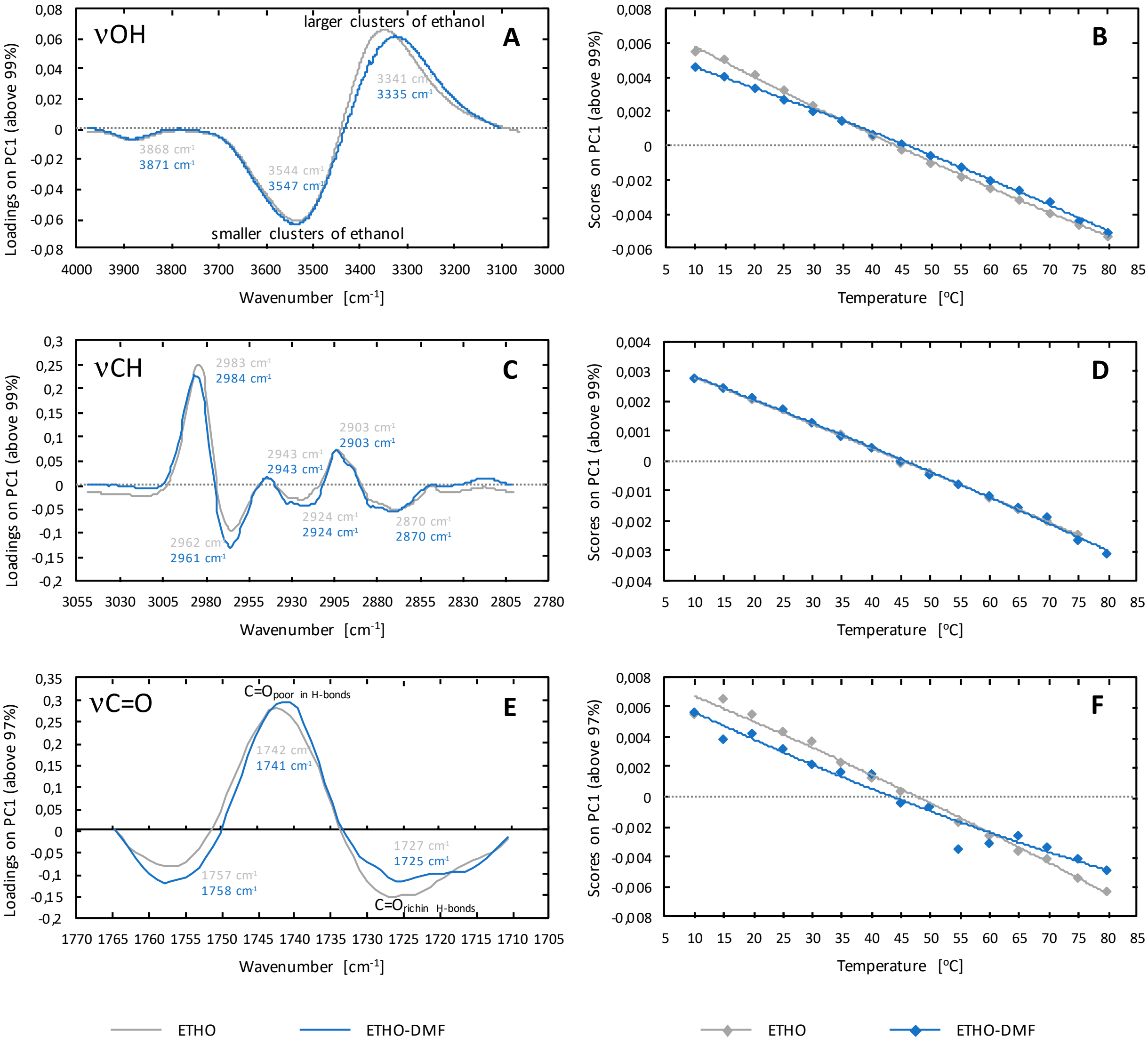

Figure S1. Loading (A, C and E) and scores (B, D and F) plots derived from PCA calculations of the νOH (A, B), νCH (C, D) and νC=O regions for ETHO (grey line) and ETHO-$DMF_{0.5}$ (blue line) samples.

In each case, a reduction of multidimensional data set to one main component was observed. Almost all changes in the total absorption (from 97 to 99 %) were attributed to this first principal component (PC1). During PCA calculations both score and loading plots were generated, see Figure S1. Similar values of scores on PC1 means high similarities between spectra measured at different temperatures. Plots of corresponding loadings show how wavenumbers are related to each other. A high value of loadings means a high influence of absorption changes at a selected wavenumber on the PC1 process. Score and loading plots derived from PCA calculations for the νC=O region of FTIR spectra of ETHO and ETHO-DMF$_{0.5}$ measured at a variety of different temperatures are shown in Figure S1 E and F. A typical behaviour of many different lipid membranes is the positioning of higher number of polar solvent molecules at the interface region of lipid membrane, as the temperature increases [1,2]. As a consequence, the number of H-bonds formed between lipid C=O groups and OH groups of solvent molecules increases as a function of temperature. As shown in Figure S1 E and F, the interface region of lipid membrane of ethosomes under study was also sensitive to changes in temperature. The positive loading peak, which corresponds to lower temperatures, with a maximum centered at higher-wavenumber range (around 1742 cm^{-1}) was attributed to the stretching vibrations of C=O groups which are poorly occupied by polar solvent molecules (Figure S1 E). The negative loading peak, which corresponds to higher temperatures, with a maximum centered at lower-wavenumber range (around 1727 cm^{-1}) was assigned to the stretching vibrations of C=O groups highly occupied by polar solvent molecules (Figure S1 E). These mentioned above loading peaks were present in both ETHO and ETHO-DMF$_{0.5}$. The linear temperature-dependent evolution of scores, shown in Figure S1 F, indicates that the number of solvent molecules, that occupy the C=O groups in the interfacial membrane region, increases linearly with a rise in temperature in both ETHO and ETHO-DMF$_{0.5}$.

Score and loading plots derived from PCA calculations for the νCH region of FTIR spectra of ETHO and ETHO-DMF$_{0.5}$ measured at a variety of different temperatures are shown in Figure S1 C and D. According to literature data [1], the νCH bands are vibrational indicators of changes in conformational order of CH groups in hydrocarbon chains. Acyl chain rotational freedom in membranes of both ETHO and ETHO-DMF$_{0.5}$ has the same linear evolution as a function of increasing temperature (Figure S1 C and S1 D).

The score and loading plots derived from PCA calculations for the third vibrational region under study (assigned to νOH bands) of ETHO and ETHO-DMF$_{0.5}$ measured at a variety of different temperatures are shown in Figure S1 A and B. The linear temperature-dependent evolution of scores derived from PCA calculations on the νOH region indicates that under a rise in temperature, a reorganization in solvent molecules takes place. In water-ethanol mixture, representing the dispersing phase of ETHO and ETHO-DMF$_{0.5}$, the ethanol molecules can aggregate into clusters with different size and morphology. According to literature data [3], a shift in position of νOH band indicates a significant rebuilding in the molecular structures of solvent molecules. The formation of larger clusters with ethanol molecules is accompanied by a decrease of νOH band wavenumber [3]. On the other hand, the formation of smaller clusters from alcohols induces a high-wavenumber shift of the νOH band [3]. The positive loading peak assigned to νOH vibrations (see Figure S1 A), with a maximum centered at lower-wavenumber range, indicates the formation a larger alcohol clusters in ETHO and ETHO-DMF$_{0.5}$ under lower temperatures. These clusters reorganize into smaller ones under the influence of temperature increase, resulting in a shift of νOH band to higher wavenumbers. It should be also mentioned that an increase in temperature can change the force of H-bonds formed between the solvent molecules. Thus, in interpretation of changes in νOH bands this aspect should be considered too.

S2. Kinetic Study of the DMF release

To investigate the kinetics and mechanisms governing DMF release through PTFE membranes, the following kinetic models were applied:

(1) The zero-order model, used for pharmaceutical dosage forms that do not disintegrate, characterized by a very slow drug release, expressed by the equation:

$$Qt = Q_0 + k0t \qquad (S1)$$

where Qt is the amount of dissolved drug at time t, Q_0 is the initial amount of drug in the solution usually (Q_0 = 0) and k_0 is the constant of zero-order release.

(2) The first-order model, used to describe the absorption and release of water soluble drugs from porous matrices, according to the equation:

$$Log_{Qt} = Log_{Q0} - k_1\ 2.303\ t \qquad (S2)$$

where Qt is the amount of dissolved drug at time t, Q_0 is the initial amount of drug in the solution, and k_1 is the constant of first-order release.

(3) The Higuchi model, widely used to describe the release of soluble and sparingly soluble drugs in aqueous media, from various semi-solid and/or solid matrices, expressed by the equation:

$$Qt = kHt\ 1/2 \qquad (S3)$$

where kH is the Higuchi dissolution constant, and Qt and t are the parameters described previously.

(4) The Korsmeyer-Peppas model, a generalised model of the Higuchi equation, allowing to explain drug delivery mechanisms characterized by both erosion and/or dissolution of the matrix. This model is usually used to describe the drug release from polymer systems. The related equation is:

$Mt/M\infty = krt\ n$ (5)

where $Mt/M\infty$ is the fraction of drug released at time t; kr is the release constant, characteristic for the polymer–drug interactions, n is the diffusion exponent, describing the release mechanism.

n = 0.5, indicates a Fickian type (case I) release mechanism, the equation is equal to the Higuchi model,

0.5 < n < 1.0 indicates an anomalous (non-Fickian) release mechanism,

n = 1.0 indicates a release mechanism similar to a zero-order release,

n > 1.0 (Super Case II transport) indicates a drug release mechanism dependent on the relaxation of the polymer chains in the matrix, from a vitreous state to a relaxed state rubber type [4,5].

Figure S2 reports the fitting to the different kinetic models of DMF release data from ETHO-$DMF_{0.5}$, EG-$DMF_{0.5}$, and G-$DMF_{0.5}$.

Notably, R^2 and n values were considered to evaluate the DMF release mechanisms.

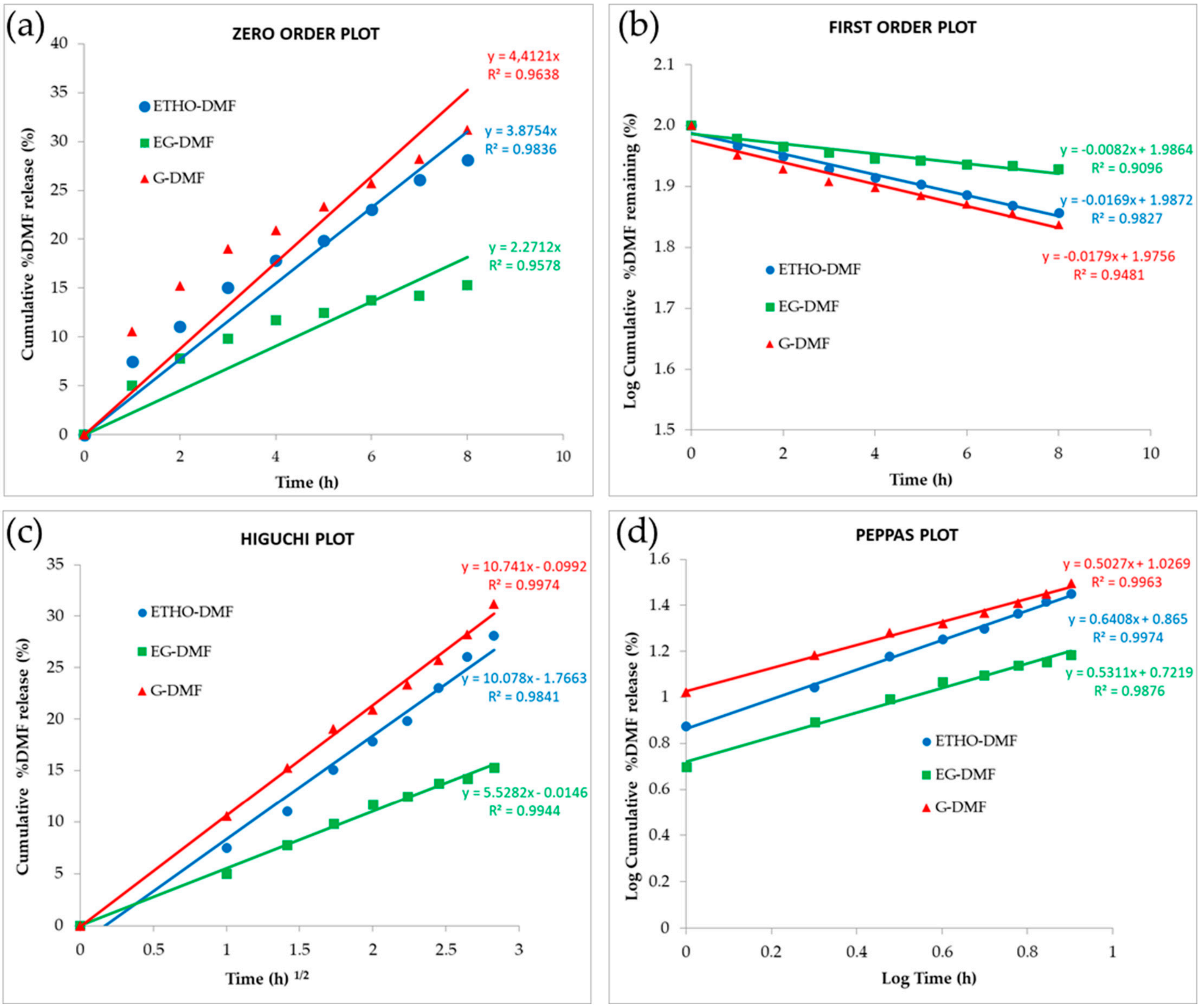

Figure S2. Fitting of DMF release data to zero-order (a), first order (b), Higuchi (c) and Korsmeyer-Peppas (d) kinetic models for the indicated formulations [4].

References

1. Cieślik-Boczula, K.; Szwed, J.; Jaszczyszyn, A.; Gasiorowski, K.; Koll, A. Interactions of Dihydrochloride Fluphenazine with DPPC Liposomes: ATR-IR and ^{31}P NMR Studies. *J. Phys. Chem. B* **2009**, *113*, 15495–15502, doi:10.1021/jp904805t.

2. Ciesik, K.; Koll, A.; Grdadolnik, J. Structural Characterization of a Phenolic Lipid and Its Derivative Using Vibrational Spectroscopy. *Vibrational Spectroscopy* **2006**, *41*, 14–20, doi:10.1016/j.vibspec.2005.12.004.

3. Golub, P.; Doroshenko, I.; Pogorelov, V.; Sablinskas, V.; Balevicius, V.; Ceponkus, J. Temperature Evolution of Cluster Structures in Ethanol. *Dataset Papers in Physics* **2013**, *2013*, 1–4, doi:10.1155/2013/473294.

4. Siepmann, J.; Siepmann, F. Modeling of Diffusion Controlled Drug Delivery. *J Control Release* **2012**, *161*, 351–362, doi:10.1016/j.jconrel.2011.10.006.

5. Salamanca, C.; Barrera-Ocampo, A.; Lasso, J.; Camacho, N.; Yarce, C. Franz Diffusion Cell Approach for Pre-Formulation Characterisation of Ketoprofen Semi-Solid Dosage Forms. *Pharmaceutics* **2018**, *10*, 148, doi:10.3390/pharmaceutics10030148.
